# Comparison of Additive Manufacturing and Injection Molding of Biocomposites Reinforced with Alkali-Treated Wood Flour Derived from Recycled Wooden Pallets

**DOI:** 10.3390/polym17152004

**Published:** 2025-07-22

**Authors:** Mehmet Demir, Nilgül Çetin, Nasır Narlıoğlu

**Affiliations:** 1 Department of Forest Industrial Engineering, Faculty of Forestry, İzmir Kâtip Çelebi University, 35620 İzmir, Türkiye; 2 Department of Biocomposite Engineering, Graduate School of Natural and Applied Sciences, İzmir Kâtip Çelebi University, 35620 İzmir, Türkiye

**Keywords:** additive manufacturing, biocomposites, biodegradable polymers, forest products, injection molding, particle-reinforced composites, PLA, wood recycling

## Abstract

Biodegradable polymer composites offer promising alternatives to petroleum-based plastics, supporting the principles of a zero waste and circular economy. This study investigates the reinforcing potential of alkali-treated wood flour derived from recycled pine (*Pinus brutia* Ten.) and poplar (*Populus alba* L.) waste wooden pallets in poly(lactic acid) (PLA) biocomposites. Wood flour was initially recovered through grinding and screening during recycling, followed by alkali treatment via a green chemistry approach to enhance interfacial bonding with the PLA matrix. The impact of alkali concentration and two fabrication methods—additive manufacturing (AM) and injection molding (IM)—on the properties of developed biocomposite materials was assessed through mechanical, physical, morphological, and thermal analyses. IM samples outperformed AM counterparts, with the IM PLA containing 30 wt% wood flour (alkali-treated with 10% solution) showing the highest mechanical gains: tensile (+71.35%), flexural (+64.74%), and hardness (+2.62%) compared to untreated samples. Moreover, the AM sample with 10 wt% wood flour and 10% alkali treatment showed a 49.37% decrease in water absorption compared to the untreated sample, indicating improved hydrophobicity. Scanning electron microscopy confirmed that alkali treatment reduced void content and enhanced morphological uniformity, while thermal properties remained consistent across fabrication methods. This work introduces a green composite using non-toxic materials and treatments, facilitating eco-friendly production aligned with zero waste and circular economy principles throughout the manufacturing lifecycle.

## 1. Introduction

By 2023, global forest cover declined by 6.37 million hectares, exceeding the 2030 deforestation target by nearly 50% [1]. This alarming depletion highlights the urgent need to recycle wood waste, particularly from high-volume sources such as wooden pallets. The global wood recycling market is projected to grow by approximately 44% by 2032, driven by rising demand for sustainable materials and circular economy practices [2]. To support this transition, it is essential to develop integrated strategies for utilizing recyclable wood biomass in bio-based product manufacturing with careful consideration of economic, environmental, and societal dimensions. Efficient resource management and carbon footprint reduction can broaden application areas and improve the performance of biomaterials. Establishing standardized protocols for biological waste utilization remains critical to ensuring long-term sustainability [3,4]. Moreover, growing concerns over plastic pollution and climate instability further reinforce the strategic importance of innovative wood reuse [5].

Wooden pallets are widely used in the global supply chain, with millions entering circulation annually. However, when these pallets reach the end of their service life, they are often discarded or recycled [6,7,8]. Discarded wooden pallets serve as a valuable raw material source for producing high-performance biocomposite materials [9]. This potential motivates increased research into pallet recycling and valorization.

Natural particle-based lignocellulosic is a complex biological material primarily composed of cellulose, hemicellulose, and lignin, with trace amounts of extractives [10]. Lignocellulosic particle-reinforced biocomposites are valued for their biodegradability, recyclability, high strength, low toxicity, affordability, and ease of processing, making them suitable for applications from packaging to medical uses [11]. A key factor affecting their properties is the interfacial adhesion between lignocellulosic particles and polymer matrices [12]. Alkali treatment effectively promotes fibrillation and surface roughening of fibers, thereby reinforcing mechanical interlocking at the fiber–matrix interface [13,14].

Additive manufacturing (AM) or 3D printing builds parts layer-by-layer, enabling complex, customized designs with minimal waste. It offers greater design flexibility, rapid prototyping, and cost reductions compared to traditional subtractive methods. Originally limited to prototyping, AM is now an established industrial process [15,16]. Advances in AM materials include polymers, composites, wood-based powders, and biodegradable alternatives, transforming 3D printing materials [17,18]. Poly(lactic acid) (PLA), a biodegradable biopolymer widely used in AM, combines mechanical strength, biocompatibility, and easy processing with renewability and eco-friendly degradation. Its compatibility with AM supports circular, sustainable manufacturing [19,20]. Injection molding (IM) remains a cost-effective, scalable method for mass-producing bio-based plastics like PLA due to its speed and product quality [21]. Comparative analysis of AM and IM products is essential to elucidate their distinct advantages and limitations across varying material parameters.

As highlighted by Pilipović et al. [22], polymer composite materials have attracted considerable attention in recent years owing to their excellent mechanical properties and low density, both of which have driven their expanding application across various engineering sectors. Despite the limited number of studies on using waste wooden pallet particlesin polymer matrices, existing research indicates encouraging outcomes. For example, Stark [9] observed improvements in tensile and flexural strength in polypropylene (PP) composites reinforced with particlesderived from pallet waste. Further, Stark and Rowlands [23] reported significant enhancements in modulus values under similar conditions. Sommerhuber et al. [24] demonstrated that high-density polyethylene (HDPE) composites incorporating up to 30% pallet-derived particlesexhibited improved performance, suggesting the feasibility of large-scale adoption. In a related study, Basalp et al. [25] confirmed that recycled wood–plastic composite (r-WPC) formulations produced from post-consumer plastics and pallet waste are suitable feedstocks for industrial composite applications, aligning well with circular economy goals. Similarly, Iždinský et al. [26] highlighted both environmental and economic benefits of incorporating recycled spruce pallets into biocomposite systems. Despite increasing interest, pallet-derived wood flour composed mainly of pine (*Pinus* spp.) and poplar (*Populus* spp.) has not yet been explored in bio-plastics or evaluated across key processing methods such as AM and IM.

Demir et al. [27] demonstrated that waste pallets derived from *Pinus brutia* Ten. and *Populus alba* L., due to their high cellulose content (~48%), remain effective biocomposite reinforcements even after extended outdoor exposure, thus promoting sustainable waste valorization. Within the broader context of circular economy and zero-waste principles, various secondary lignocellulosic by-products—such as wooden poles, wood flour, sawdust, pruning residues, and hemp fibers—have demonstrated promising potential as renewable feedstocks for the development of biocomposites, particularly those reinforced with biodegradable PLA-based matrices [28,29,30,31,32,33,34,35]. Waste-based biocomposites offer sustainable, cost-effective solutions crucial for addressing environmental and economic challenges while driving innovation.

This study investigates the use of alkali-treated wood flour extracted from waste pallets of *Pinus brutia* Ten. (Turkish red pine) and *Populus alba* L. (white poplar) as reinforcing agents in PLA-based biocomposites. The biocomposites were produced via both AM and IM, enabling a direct comparison of their respective advantages and constraints. Although substantial progress has been made in developing wood waste-reinforced PLA composites, the systematic incorporation of alkali-treated pallet particles into bioplastics through both AM and IM has not yet been addressed in the literature. In particular, the influence of the specific wood species used in this study—linked to their differing chemical compositions—on composite performance has not been comparatively studied. This research aims to bridge this gap by comprehensively evaluating the physical, mechanical, morphological, and thermal behavior, interfacial adhesion, and processing potential of PLA-based biocomposites reinforced with alkali-treated pallet wood flour, advancing sustainable material development. The study offers novel comparative data on how two wood types and manufacturing methods influence biocomposite structure and properties, advancing sustainable bio-based materials.

## 2. Materials and Methods

### 2.1. Materials

Waste wooden pallets from *Pinus brutia* Ten. (Turkish red pine) and *Populus alba* L. (white poplar) were collected from İzmir Kâtip Çelebi University (İzmir, Türkiye). PLA Bio-Flex^®^ F 7510 (FKuR Plastics Corp., Willich, Germany) was employed as the polymer matrix for the biocomposite production. Sodium hydroxide (NaOH) was used for surface modification and for the chemical characterization of wood flours. Additional chemicals used in the characterization process included acetic acid (CH_3_COOH), acetone, ethanol, sodium chlorite (NaClO_2_), and toluene. All were purchased from Merck (Darmstadt, Germany).

### 2.2. Methods

Pine and poplar wood were ground with an LKD 100 grinder (Loyka, Akyol Ticaret, İstanbul, Türkiye). The ground wood particles, finer than 1 mm, were then sieved using an ESM 200 sieve shaker (Loyka, Akyol Ticaret, İstanbul, Türkiye), passing through a 60-mesh sieve. Particles retained by the 80-mesh sieve were separated and collected [36,37]. The separated wood flour was dried thoroughly at 103 ± 2 °C prior to biocomposite production.

#### 2.2.1. Particle Size Analysis of Wood Flour

Particle size distribution (PSD) was measured using a Malvern Mastersizer 3000 laser diffraction analyzer (Malvern Instruments, Ltd., Malvern, UK). A dry dispersion method with 4-bar air pressure and 15% feed rate ensured effective dispersion and 5% obscuration. PSD measurements were performed five times, and averages reported [38].

#### 2.2.2. Surface Modification of Wood Flour with Alkaline Treatment

The fillers underwent alkali treatment by immersing separate batches in NaOH solutions at concentrations of 2.5%, 5%, and 10%, each for 6 h at room temperature. Each group was treated independently to evaluate the influence of different alkali concentrations on surface roughness and adhesion to the polymer matrix. This treatment roughens the surface, improving its adhesion to the polymer matrix. Subsequently, the fillers were rinsed with distilled water to remove residual NaOH and neutralized using a 2% acetic acid solution. After a second rinse to eliminate any remaining acid and adjust the pH to around 7, the wood flour was dried at 80 °C for 48 h [39].

#### 2.2.3. Production of Filaments and Pellets

Wood flour was incorporated into PLA polymer at 10, 20, and 30 wt% concentrations for the production of both filaments (for AM) and pellets (for IM) (Table 1). Prior to blending, PLA was dried at 80 °C for 6 h.

For filament production, biocomposite strands were extruded using a microprocessor-controlled co-rotating twin-screw extruder equipped with five heating zones, a nozzle diameter of 1.75 ± 0.1 mm, and an L/D ratio of 20. The extrusion was conducted at 100–170 °C with a screw speed of 50 rpm, followed by cooling and spooling.

Melt compounding for pellet production was performed using a twin-screw extruder, with the extrusion temperature profile along the screw set as follows: 100 °C (feed zone), 160 °C, 170 °C, 180 °C, 180 °C, 170 °C, and 160 °C (die zone). The extrudate was quenched in cold water and then pelletized.

Subsequently, the processed filaments and pellets were subjected to a second thermal step—AM or IM, respectively—to fabricate the final test specimens. This dual thermal exposure is acknowledged as a potentially influential factor on the thermal and mechanical properties of the resulting biocomposites.

#### 2.2.4. AM of Test Samples

The AM process was conducted using an open-source Sigma 3D printer, operated via Repetier-Host software (version 2.3.2, Hotworld GmbH, Willich, Germany). The following printing parameters were selected: 100% infill density, a print speed of 50 mm/s, a layer thickness of 0.3 mm, and a raster angle of ±45°. The extruder nozzle was maintained at 190 °C, and the heated bed was set to 55 °C. Optimized parameters enabled precise fabrication of ASTM-standard samples, with five replicates for mechanical and three for physical tests, all meeting performance criteria.

#### 2.2.5. IM of Test Samples

The extruded neat and filled granules were dried at 60 °C for 4 h and then molded into test samples using an IM machine model FTM 1580 (Manisa, Türkiye), following the ASTM D3641-21 standard [40]. The molding temperature was set between 170–180 °C, and the injection pressure was adjusted to 110–120 bar. Fine-tuned settings ensured precise fabrication of ASTM-compliant samples, with five specimens for mechanical and three for physical tests.

#### 2.2.6. Determination of the Mechanical Properties

The tensile (Type IV) and flexural strengths of the biocomposite test samples were determined using an MTS Criterion^®^ Series Model 45 (Prairie, MN, USA) universal testing machine, following the ASTM D638-22 (tensile) [41] and ASTM D790-17 (flexural) [42] standards. Shore D hardness tests were conducted with a digital hardness measurement device, LXD-D (Loyka, İstanbul, Türkiye), following the ASTM D2240-15 [43]. Prior to all tests, the samples were conditioned for 7 days at 50 ± 5% relative humidity and 23 ± 2 °C. Each parameter group was tested with five replicates to ensure experimental robustness.

#### 2.2.7. Determination of the Physical Properties

In order to determine the water resistance properties of the biocomposite test samples, the samples were immersed in distilled water for a period of 1 month. Prior to this test, the samples were conditioned for 48 h at 50 ± 5% relative humidity and 23 ± 2 °C, following the ASTM D570-22 standard [44] for water absorption testing. Density for all formulations was determined by weighing samples in air and water using a density determination apparatus with precision scales following the ASTM D792-20 standard [45]. Measurements were conducted in triplicate to ensure reliability.

#### 2.2.8. Morphological Characterization by Scanning Electron Microscopy (SEM)

The SEM images were captured to examine the interaction between the wood flour and the PLA polymer matrix. Both untreated and alkali-treated wood flour, along with the fracture surfaces of the biocomposites, were gold-coated using an EM ACE200 sputter coater (Leica Microsystems, Wetzlar, Germany), and subsequently scanned using a Gemini 500 SEM (ZEISS, Baden-Württemberg, Germany). SEM analysis was conducted on fracture surfaces of tensile specimens with the highest and lowest recovery to reveal microstructural causes of performance variation.

#### 2.2.9. Determination of the Thermal Properties

Thermal analysis was performed on tensile specimens with the highest and lowest recovery rates to assess their thermal stability, degradation behavior, phase transitions, and structural changes. The thermogravimetric analysis (TGA) of the produced wood/PLA composite was performed using a Q600 TGA-STD (TA Instruments, New Castle, DE, USA). TGA measurements were conducted in an inert nitrogen atmosphere with a flow rate of 50 mL/min, at a heating rate of 10 °C/min, a temperature range of 25 to 600 °C.

Differential scanning calorimetry (DSC) analysis of the samples was conducted using a DSC Q2000 thermal analysis system (TA Instruments, New Castle, DE, USA). Samples weighing between 5 and 10 mg were subjected to a heating program in a nitrogen atmosphere at a rate of 10 °C/min, from room temperature to 250 °C, with a flow rate of 50 mL/min. Thermal transitions, including *T_g_*, *T_c_*, *T_m_*, and *T_cc_* were identified from DSC heating and cooling curves through characteristic endothermic and exothermic peaks. Crystallinity (*X_cr_*) was then calculated using Equation (1):

(1) $$X_{cr}=\left(\;\frac{{\Delta H}_{m}-{\Delta H}_{cc}}{{{\Delta H}_{m}}^{0}\;\times \;w_{PLA}}\right)\;\times \;100$$

Δ*H_m_* and Δ*H_cc_* represent the melting and cold crystallization enthalpies from DSC, respectively. Δ*H_m_*^0^ is the theoretical enthalpy for fully crystalline PLA (93 J·g^−1^), and *w_PLA_* is the PLA mass fraction in the composite [35,46].

#### 2.2.10. Chemical Characterization by Fourier Transform Infrared (FT-IR) Spectroscopy

A Nicolet™ iS50 FT-IR spectrometer (Thermo Fisher Scientific Inc., Waltham, MA, USA) was used to analyze potential chemical bonds in both untreated and alkali-treated wood flours and their composites. The analysis was conducted at room temperature using the standard attenuated total reflectance (ATR) method. The FT-IR spectra for each sample were recorded in the range of 400 cm^−1^ to 4000 cm^−1^ using an IR spectrophotometer to ensure high-resolution spectral data. FT-IR spectroscopy was used to examine the chemical differences and interactions in tensile samples with the highest and lowest recovery ratios. Additionally, the chemical composition of alkali-treated waste lignocellulosic materials was determined in accordance with TAPPI standards for sample preparation (T 257) [47], moisture content (T 550) [48], ash (T 211) [49], extractives (T 204) [50], holocellulose (chlorite method; T 222) [51], and α-cellulose (T 203) [52].

#### 2.2.11. Statistical Analysis of Mechanical Test Results

The statistical significance of the mechanical test results was evaluated using analysis of variance (ANOVA), followed by the Tukey’s test, performed with SPSS software (version 22, IBM, Armonk, NY, USA). Statistically significant differences between test samples are represented by distinct lowercase letters (*p* < 0.05) in the outcome graphs of the mechanical and physical tests.

## 3. Results and Discussion

### 3.1. PSD of Wood Flour

The particle sizes obtained exhibit substantial variation as a function of raw material type. Due to the anisotropic geometry of wood particles, characterized by varying aspect ratios, the PSD curves become increasingly broader as the particle size increases from 100 µm to 400 µm, reflecting a higher degree of morphological heterogeneity [53,54,55].

In the cumulative PSD, the 10th, 50th, and 90th percentiles are represented as D10, D50 (the median particle size), and D90, respectively, based on the volume fraction, where the D50 value specifically denotes the particle diameter below which 50% of the cumulative volume of the sample is distributed [56].

**Figure 1:**
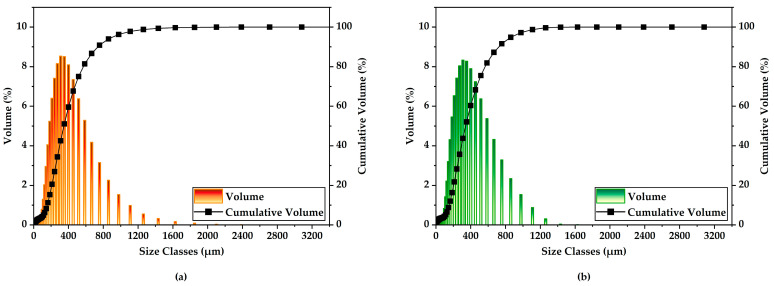
PSD of the wood flour; (**a**) pine and (**b**) poplar.

The PSD profiles and the corresponding analytical results for wood flour are presented in Figure 1 and summarized in Table 2. The D50 values for pine and poplar wood particles were determined to be 346 and 341 µm, respectively.

### 3.2. Chemical Properties of Fillers

**Figure 2:**
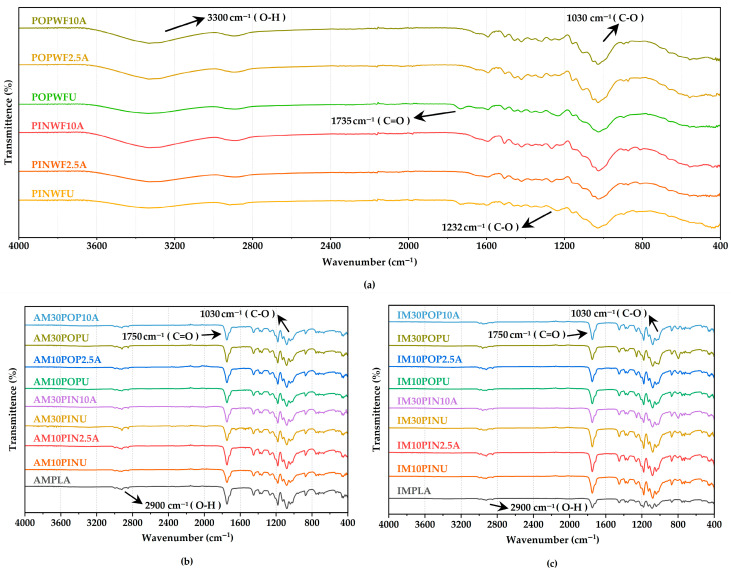
FT-IR spectrums of wood flour (**a**), AM (**b**), and IM (**c**).

FT-IR spectroscopy was employed to investigate the chemical composition and potential interfacial interactions among untreated and NaOH-treated waste wood flour, the neat PLA, and their corresponding biocomposites (Figure 2). The present findings show a clear congruence with previously reported data, thereby reinforcing the reproducibility and reliability of the observed outcomes [57,58,59,60,61].

In our previous study [27], we characterized the chemical compositions of wood flour extracted from waste wooden pallets made of Turkish red pine and white poplar, as detailed in Table 3. The results demonstrated that these waste-derived wood flour contain notably high concentrations of primary chemical components. The present study builds upon these findings. Additionally, the chemical compositions of alkali-treated wood flours were analyzed in this study and included in the same table for direct comparison. The alignment between the current findings and previously reported data reinforces the reproducibility and reliability of the results [62].

FT-IR analysis revealed clear changes in the chemical structure of wood flour following alkali treatment. A characteristic absorption band corresponding to C–O stretching vibrations was distinctly observed at approximately 1030 cm^−1^. A broad and intense absorption band between 2900 and 3300 cm^−1^, attributed to O–H stretching vibrations originating from the D-pyranose glucose units in the cellulose backbone, was observed in all spectra. Absorption bands at 1735 cm^−1^ and 1232 cm^−1^, associated with C=O stretching of aldehyde carbonyl groups, indicated the presence of lignin and hemicellulose. As illustrated in Figure 2a, these bands disappeared in the alkali-treated wood flour, indicating the effective removal of lignin and hemicellulose by NaOH treatment. Similar findings have been reported in previous studies following NaOH treatment of lignocellulosic particles [57,58,59,60,61], and the findings in the present work further support this observation.

However, FT-IR spectra of biocomposites reinforced with untreated and alkali-treated wood flour exhibited no significant differences, suggesting that while alkali treatment modifies the chemical composition of the filler, it does not lead to the formation of new detectable interfacial chemical bonds between the wood flour and the PLA matrix (Figure 2b,c). Instead, the enhanced interfacial performance is more plausibly attributed to improved mechanical interlocking, as supported by morphological and mechanical characterizations discussed elsewhere in this study.

### 3.3. Mechanical Properties of Biocomposites

#### 3.3.1. Tensile Test Results

PLA-based biocomposite specimens produced via AM with varying raster orientations exhibited slightly lower tensile strength than their IM counterparts. This difference is primarily attributed to void formation during the layer-by-layer deposition process [21,63]. Overall, alkali pre-treatment of lignocellulosic particles significantly improved the tensile strength and modulus of the biocomposites by enhancing interfacial adhesion between PLA and the particles and reducing interfacial voids [64,65,66,67,68,69]. These findings align with previously published data, indicating consistency in the observed trends [21,63,64,65,66,67,68,69].

**Figure 3:**
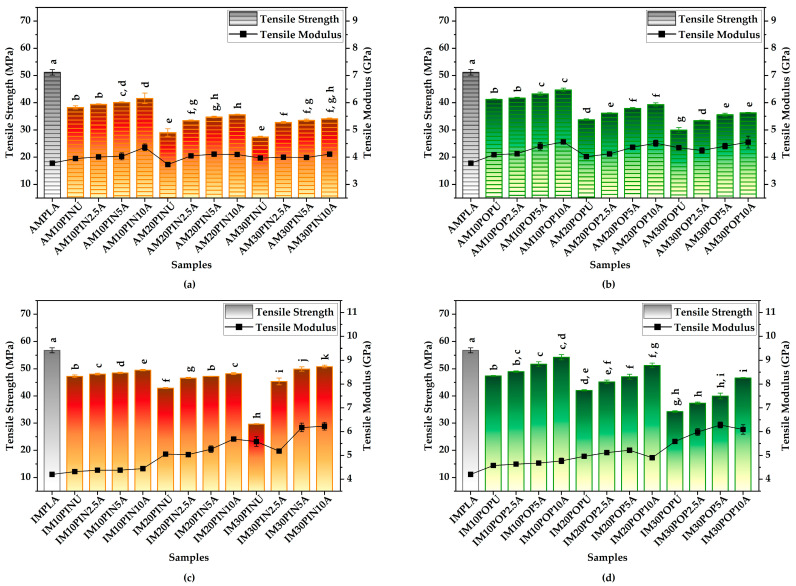
Tensile strength and modulus of AM (**a**,**b**) and IM (**c**,**d**); red = pine, green = poplar; lowercase letters = significant differences (*p* < 0.05).

Among the AM samples reinforced with pine wood flour, AM30PIN10A exhibited the greatest improvement in tensile strength, showing a statistically significant 24.18% increase compared to its untreated counterpart (from 27.43 MPa to 34.07 MPa; *p* < 0.001), as illustrated in Figure 3a,b. Similarly, AM30POP10A showed the highest enhancement among poplar wood flour-reinforced AM samples, with a 20.89% increase (from 30.00 MPa to 36.27 MPa; *p* < 0.001). In contrast, samples with lower wood flour content, such as AM10PIN2.5A and AM10POP2.5A, demonstrated only modest improvements of 3% (from 38.22 MPa to 39.37 MPa; *p* > 0.05) and 1.21% (from 41.20 MPa to 41.70 MPa; *p* > 0.05), respectively.

In terms of tensile modulus, similar trends were observed. AM10PIN10A showed the highest improvement (10.30%) among pine wood flour-reinforced AM samples (from 3.95 GPa to 4.36 GPa; *p* < 0.001), while AM20POP10A exhibited a statistically significant increase (12.21%) among poplar wood flour-based samples (from 4.02 GPa to 4.5 GPa; *p* < 0.001). The AM-fabricated neat PLA sample (AMPLA) recorded the lowest tensile modulus, at 3.78 GPa (Figure 3a,b).

The effectiveness of alkali treatment in IM samples. Among pine-reinforced IM biocomposites, IM30PIN10A exhibited a 71.35% increase in tensile strength following treatment (from 29.67 MPa to 50.83 MPa; *p* < 0.001). Similarly, the poplar wood flour-reinforced IM30POP10A showed the highest tensile strength among its group, reaching 46.63 MPa, which corresponds to a statistically significant 36.16% improvement over its untreated counterpart (34.25 MPa; *p* < 0.001). Although the IM-fabricated neat PLA (IMPLA) sample exhibited the highest absolute tensile strength (56.77 MPa), the relative enhancements through wood flour treatment underscore the crucial role of surface modification. IM samples with lower wood flour content, such as IM10PIN2.5A and IM10POP2.5A, exhibited limited tensile strength increases of 1.78% (from 47.23 MPa to 48.07 MPa; *p* > 0.05) and 3.36% (from 47.37 MPa to 48.96 MPa; *p* < 0.05), respectively, as shown in Figure 3c,d.

The tensile modulus of IM biocomposites increased progressively with higher contents of untreated wood flour, indicating enhanced stiffness despite potential reductions in tensile modulus due to interfacial weaknesses, as illustrated in Figure 3c,d. However, when the same wood flour content was treated with 10% NaOH, a further improvement was observed. Notably, the pine wood flour-based formulation IM30PIN10A, incorporating 30 wt% alkali-treated wood flour, achieved a tensile modulus of 6.23 GPa, an 11.61% increase over its untreated counterpart (5.58 GPa), which was statistically significant (*p* < 0.05). Similarly, among the poplar wood flour-reinforced biocomposites, IM30POP5A exhibited the greatest enhancement, with a 12.38% increase in tensile modulus compared to the untreated sample (rising from 5.59 GPa to 6.28 GPa; *p* < 0.05). In contrast, IMPLA recorded the lowest tensile modulus, at 4.20 GPa.

These results clearly demonstrate that alkali treatment significantly enhances the mechanical properties of PLA-based biocomposites, especially at higher wood flour contents. While IM samples generally outperform AM ones due to better layer adhesion, optimizing both wood flour treatment and processing method is key to minimizing defects and improving performance. These findings provide valuable insights for tailoring processing conditions and surface treatments to maximize the performance of biocomposites, advancing their practical use in sustainable material solutions.

#### 3.3.2. Flexural Test Results

PLA-based biocomposite specimens fabricated via AM exhibited slightly lower flexural strength and modulus than their IM counterparts, mainly due to the presence of voids and weaker layer adhesion [21]. Progressive alkali pre-treatment significantly enhanced flexural properties by strengthening particle–matrix bonding and minimizing void formation through better matrix dispersion. The results obtained in this study exhibit strong consistency with data previously reported in the literature [21,65,70].

**Figure 4:**
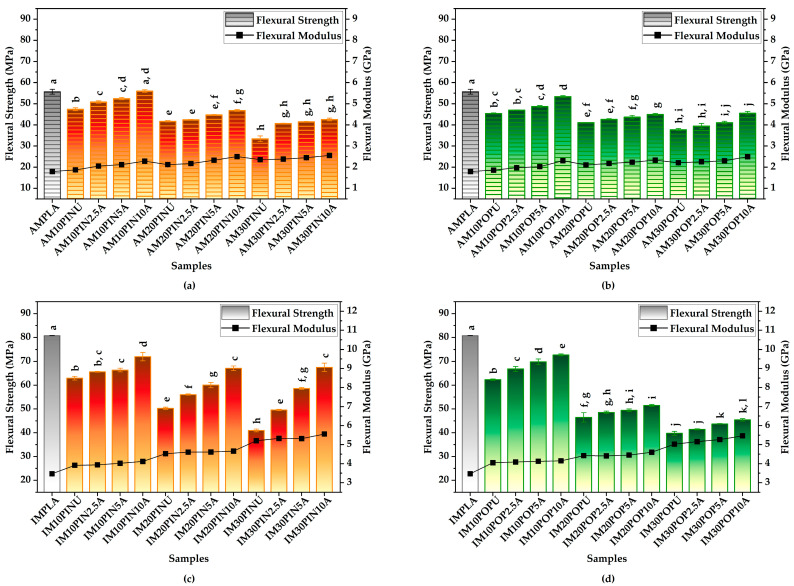
Flexural strength and modulus of AM (**a**,**b**) and IM (**c**,**d**); red = pine, green = poplar; lowercase letters = significant differences (*p* < 0.05).

As illustrated in Figure 4, neat PLA specimens produced via IM exhibited higher flexural strength than those produced via AM. However, the incorporation of untreated wood flour into the PLA matrix led to a general decrease in flexural strength in both AM and IM specimens, likely due to poor interfacial compatibility. The addition of untreated wood flour to the PLA matrix resulted in a marked enhancement in flexural modulus, with IM samples consistently exhibiting superior values compared to those fabricated via AM.

Among the AM samples reinforced with pine wood flour, AM30PIN10A demonstrated the most significant enhancement in flexural strength, achieving a statistically significant 27.29% increase compared to its untreated counterpart (from 33.43 MPa to 42.55 MPa; *p* < 0.001). Similarly, among the AM samples reinforced with poplar wood flour, AM10POP10A exhibited the highest increase, resulting in a statistically significant 17.62% increase in flexural strength relative to the untreated sample (from 45.40 MPa to 53.40 MPa; *p* < 0.001), as seen in Figure 4a,b.

As seen in Figure 4a,b, a similar increasing trend in flexural modulus was observed in the flexural modulus. Among the AM samples reinforced with pine wood flour, AM10PIN10A exhibited the highest improvement, showing a statistically significant 21.92% increase compared to its untreated counterpart (from 1.87 GPa to 2.28 GPa; *p* < 0.001).

On the other hand, as illustrated in Figure 4a,b, the most notable enhancement among the AM samples reinforced with poplar wood flour was observed in AM10POP10A, which exhibited a statistically significant 24.07% increase in flexural modulus compared to its untreated counterpart (from 1.86 GPa to 2.31 GPa; *p* < 0.001). Within this group, the AMPLA sample exhibited the lowest flexural modulus, with a value of 1.79 GPa.

Among the IM samples reinforced with pine wood flour, IM30PIN10A showed the highest improvement in flexural strength, achieving a 64.74% increase compared to its untreated counterpart (from 40.99 MPa to 67.53 MPa; *p* < 0.001), as shown in Figure 4c,d. Similarly, among the IM samples reinforced with poplar wood flour, IM10POP10A demonstrated the most significant enhancement, achieving a 16.72% increase in flexural strength relative to the untreated sample (from 62.31 MPa to 72.73 MPa; *p* < 0.05).

As seen in Figure 4c,d, the flexural modulus of IM biocomposites progressively increased with rising untreated wood flour content. The pine-wood flour-reinforced IM30PIN10A achieved a flexural modulus of 5.56 GPa, corresponding to a 6.75% enhancement compared to its untreated counterpart (5.21 GPa; *p* < 0.05). Similarly, among the poplar wood flour-reinforced biocomposites, IM30POP10A exhibited the highest enhancement, with an 8.55% increase compared to its untreated counterpart (from 5.03 GPa to 5.46 GPa; *p* < 0.05). The IMPLA sample recorded the lowest flexural modulus at 3.47 GPa.

#### 3.3.3. Hardness Test Results

The incorporation of both untreated and alkali-treated lignocellulosic particles has been demonstrated to significantly enhance the hardness of IM PLA composites. In particular, alkali treatment effectively eliminates non-cellulosic components such as hemicellulose and lignin, resulting in improved interfacial adhesion between the wood flour and the PLA matrix. This improvement minimized micro-void formation and resulted in a notable increase in Shore D hardness [71]. Conversely, the increased incorporation of untreated wood flour into 3D-printed biocomposites is consistently linked to a marked reduction in material hardness, likely due to the significant porosity induced by higher filler loading [31,72].

**Figure 5:**
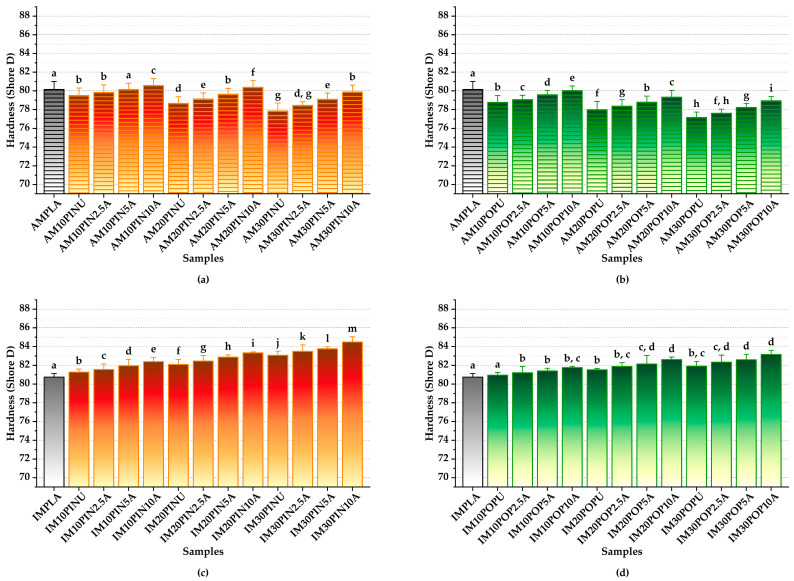
Hardness of AM (**a**,**b**) and IM (**c**,**d**); red = pine, green = poplar; lowercase letters = significant differences (*p* < 0.05).

As seen in Figure 5, neat PLA specimens fabricated via IM exhibit slightly higher Shore D hardness values compared to those produced by AM. While the incorporation of untreated wood flour into the PLA matrix enhanced the hardness in IM specimens, a reduction in hardness was observed in their AM counterparts. This reduction is primarily attributed to the presence of micro-voids and inconsistent wood flour distribution caused by the layer-by-layer nature of AM.

While the Shore D hardness of 3D-printed PLA-based biocomposites typically ranges between 70 and 80, IMPLA exhibits values exceeding 80 [71,73]. The findings of this study are in agreement with those reported in previous research [31,71,72,73].

Among the AM samples reinforced with pine wood flour, AM30PIN10A exhibited the highest gain improvement in hardness, achieving a 2.62% increase compared to the untreated counterpart (from 77.84 to 79.88; *p* < 0.05), as illustrated in Figure 5a,b. Likewise, among the AM samples reinforced with poplar wood flour, AM30POP10A demonstrated the most remarkable enhancement, achieving a 2.35% increase in hardness relative to the untreated sample (from 77.11 to 78.92; *p* < 0.05). The AMPLA exhibited Shore D hardness of 80.14 within the AM group.

As shown in Figure 5c,d, among the IM samples reinforced with pine wood flour, IM30PIN10A demonstrated the most notable rise in hardness, achieving a 1.69% increase compared to its untreated counterpart (from 83.08 to 84.48; *p* < 0.05). Similarly, among the IM samples reinforced with poplar wood flour, IM30POP10A demonstrated the most significant enhancement, achieving a 1.54% increase in hardness relative to the untreated sample (from 81.90 to 83.16; *p* < 0.05). In contrast, the IMPLA exhibited the lowest Shore D hardness of 80.74 within the IM group.

### 3.4. Physical Properties of Biocomposites

#### 3.4.1. Density Test Results

Compared to poplar (*Populus* spp.) wood, pine (*Pinus* spp.) wood exhibits significantly higher specific density values [74]. Due to inherent void formation during the AM process, 3D-printed specimens showed lower density and inferior mechanical properties compared to IM counterparts, reflecting the structural limitations of AM in producing compact and mechanically robust components [21].

The neat PLA samples exhibited relatively lower density compared to both untreated and alkali-treated PLA composites, highlighting the influence of filler incorporation and processing on material densification [75]. An inverse relationship is observed between untreated wood flour content and material density in 3D-printed WF-PLA biocomposites, with higher wood flour loadings leading to a measurable reduction in overall density [76]. Moreover, the incorporation of alkali-treated wood flour into biocomposite matrices resulted in noticeably higher density than their untreated counterparts, underscoring the role of chemical surface modification in improving interfacial compatibility and material compactness [64].

**Figure 6:**
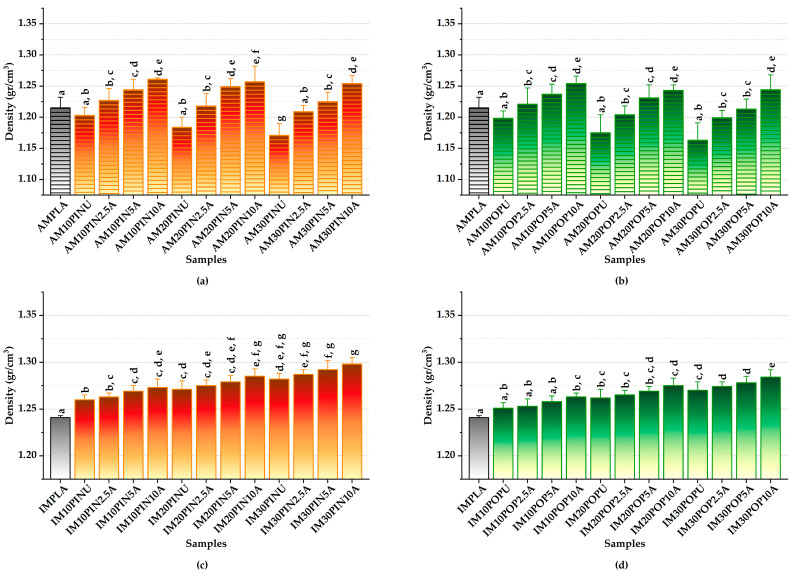
Density of AM (**a**,**b**) and IM (**c**,**d**); red = pine, green = poplar; lowercase letters = significant differences (*p* < 0.05).

IMPLA samples exhibit slightly higher density compared to their additively manufactured counterparts (Figure 6). Among the AM samples reinforced with pine wood flour, AM30PIN10A demonstrated the most significant enhancement in density, achieving a 7.09% increase compared to its untreated counterpart (from 1.17 to 1.25 gr/cm^3^; *p* < 0.05), as seen in Figure 6a,b. Similarly, among the AM samples reinforced with poplar wood flour, AM30POP10A showed the largest increase in density, achieving a 6.96% rise relative to the untreated sample (from 1.16 to 1.24 gr/cm^3^; *p* < 0.05). The AMPLA exhibited a density of 1.22 gr/cm^3^ within the AM group.

As shown in Figure 6c,d, among the IM samples reinforced with pine wood flour, IM30PIN10A demonstrated a statistically significant enhancement in density, achieving a 1.25% increase compared to its untreated counterpart (from 1.28 to 1.30 gr/cm^3^; *p* < 0.05). Similarly, among the IM samples reinforced with poplar wood flour, IM30POP10A demonstrated the highest observed increase, achieving a 1.10% increase in density relative to the untreated sample (from 1.27 to 1.28 gr/cm^3^; *p* > 0.05). The IMPLA exhibited the density of 1.24 gr/cm^3^ within the IM group.

These findings align well with those documented in prior studies, reinforcing the consistency of the observed trends [21,64,74,75,76].

#### 3.4.2. Water Absorption Test Results

The surface of the poplar wood consistently exhibits significantly lower wettability relative to the pine wood, clearly reflecting its inherently hydrophobic nature [77]. Alkali pretreatment significantly improves interfacial adhesion between lignocellulosic particles and the PLA matrix, thereby significantly reducing water absorption in treated lignocellulosic particle-reinforced biocomposites compared to their untreated counterparts [65,71,78]. Conversely, biocomposites reinforced with unprocessed wood flour exhibit considerably lower water resistance relative to those reinforced with processed wood flour. This behavior is predominantly attributed to the high density of hydroxyl (–OH) groups within unprocessed lignocellulosic particles, which impart pronounced hydrophilicity [79,80,81].

**Figure 7:**
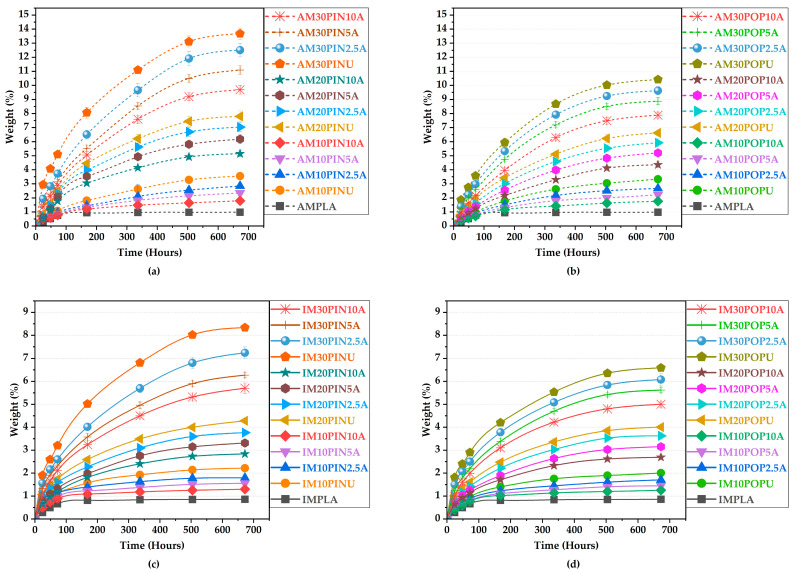
Water uptake of AM (**a**,**b**) and IM (**c**,**d**).

AM10PIN10A, among the samples reinforced with pine wood flour, exhibited a 49.37% reduction in water absorption compared to its untreated counterpart (from 3.56% to 1.80%; *p* < 0.05), indicating a significant improvement in water resistance, as illustrated in Figure 7a,b. Similarly, AM10POP10A, reinforced with poplar wood flour, achieved a 47.15% decrease in water absorption relative to the untreated sample (from 3.33% to 1.76%; *p* < 0.05). These results confirm the significant improvement in water repellency of treated biocomposites.

As shown in Figure 7c,d, IM10POP10A, among the samples reinforced with poplar wood flour, demonstrated a 41.32% reduction in water absorption relative to the untreated sample (from 2.22% to 1.30%; *p* < 0.05), clearly indicating a significant enhancement in water resistance performance. Similarly, IM10POP10A, reinforced with poplar wood flour, exhibited a 37.81% reduction in water absorption compared to its untreated counterpart (from 2.01% to 1.25%; *p* < 0.05), indicating a statistically significant improvement in water resistance.

### 3.5. Morphological Properties of Biocomposites

**Figure 8:**
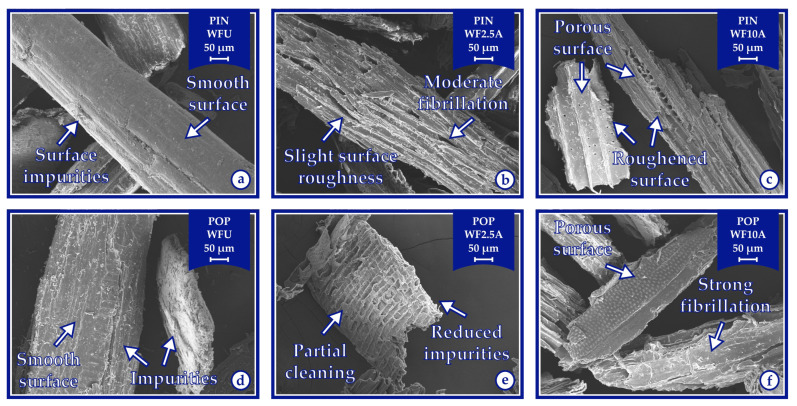
The SEM images (160× magnification) of surfaces of pine wood flour (**a**–**c**) and poplar wood flour (**d**–**f**).

The untreated wood flour exhibited a relatively smooth surface morphology, whereas alkali-treated samples showed increasingly rough and porous surfaces with higher treatment concentrations, as a result of the effective removal of surface impurities and non-cellulosic components (Figure 8).

**Figure 9:**
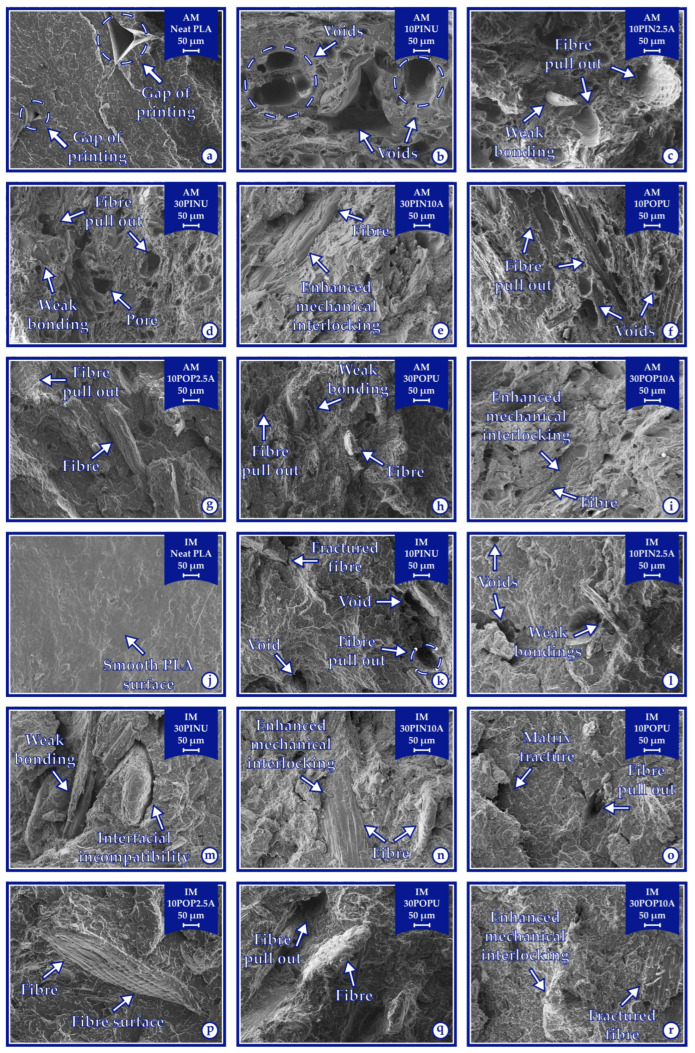
The SEM images (160× magnification) of the breaking surfaces of the additive manufactured (**a**–**i**) and injection-molded (**j**–**r**).

In the AMPLA specimen (Figure 9a), the layered architecture resulting from the printing orientation, combined with the low infill density, led to the formation of distinct interlayer voids. In contrast, the IMPLA counterpart (Figure 9j), fabricated under high-pressure conditions, exhibited a smooth and compact surface morphology, indicative of minimal or no air entrapment [82]. Moreover, the interfacial voids between the alkali-treated wood flour and the PLA matrix in the biocomposites (Figure 9e,i,n,r) were markedly reduced, demonstrating a substantially lower occurrence than those observed around untreated wood flour [83].

Figure 9 presents the cross-sectional SEM images of samples fabricated from neat PLA via AM (Figure 9a: AMPLA) and IM (Figure 9j: IMPLA), alongside unmodified wood flour-reinforced biocomposites. These biocomposites include specimens produced by AM: (b) AM10PINU, (d) AM30PINU, (f) AM10POPU, and (h) AM30POPU; and by IM: (k) IM10PINU, (m) IM30PINU, (o) IM10POPU, and (q) IM30POPU. Additionally, the figure highlights the biocomposite samples showing the most significant tensile strength improvements, including AM specimens: (e) AM30PIN10A and (i) AM30POP10A; and IM specimens: (n) IM30PIN10A and (r) IM30POP10A. In contrast, the lowest tensile strength enhancements were observed in AM specimens: (c) AM10PIN2.5A and (g) AM10POP2.5A; and IM specimens: (l) IM10PIN2.5A and (p) IM10POP2.5A.

Microstructural insights derived from SEM analysis offer compelling evidence linking the observed morphological characteristics to the variations in the specimens’ physical, mechanical, and thermal performance.

### 3.6. Thermal Properties of Biocomposites

#### 3.6.1. DSC Results

**Figure 10:**
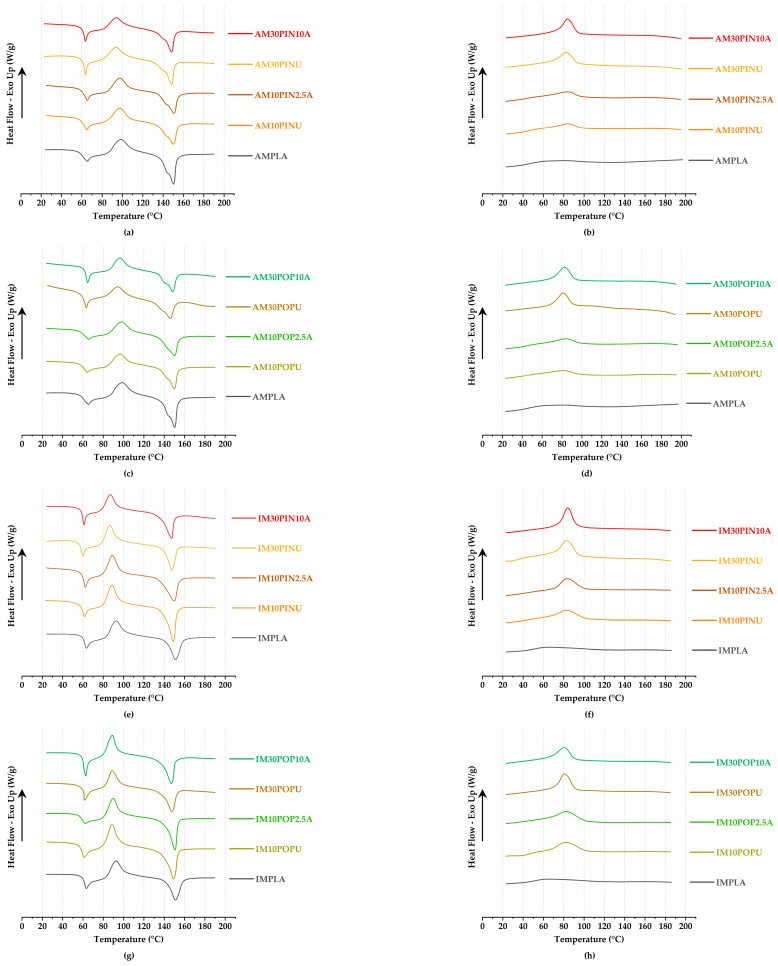
DSC curves of AM (**a**–**d**) and IM (**e**–**h**) biocomposites, compared to neat PLA.

DSC results, presented in Figure 10 and Table 4, were used to identify key thermal transitions of the wood flour and composites, including glass transition (*T_g_*), crystallization (*T_c_*), melting (*T_m_*), and cold crystallization (*T_cc_*) temperatures. *T_g_* values of neat PLA produced by AM and IM were 65.71 °C and 63.12 °C, respectively.

The influence of wood flour content and processing method on the *T_c_*, *T_g_*, *T_m_*, and *T_cc_* is clearly discernible in the DSC profiles. Results indicate that filler type, content, and fabrication method critically influence the thermal properties and crystallinity of PLA-based biocomposites.

Analysis of the thermal crystallization profiles clearly reveals that neat PLA exhibits markedly sluggish crystallization kinetics, characterized by an exceptionally broad and diffuse crystallization peak [84]. The incorporation of wood flour into PLA affects its thermal transitions, with wood flour type and treatment playing a key role. Wasti et al. [85] and Kamarudin et al. [86] observed comparable thermal behavior trends, including reductions in glass transition and crystallization temperatures, as untreated wood flour was added to the matrix material. Notably, alkali-treated wood flour slightly improved crystallinity, highlighting the impact of surface treatment on thermal behavior.

DSC analysis revealed a slight increase in crystallinity (*X_cr_*) for biocomposites with alkali-treated wood flour that exhibited the highest tensile strength. Specifically, *X_cr_* increased from 13.37% to 13.54% in AM10PIN2.5A (an increase of 1.27%) and from 13.33% to 13.50% in AM10POP2.5A (an increase of 1.28%) compared to their untreated counterparts. These values remained slightly below that of the AMPLA, which exhibited an *X_cr_* of 14.40%. Likewise, IM samples IM30PIN10A and IM10POP2.5A showed minor increases of 1.12% (from 10.70% to 10.82%) and 1.10% (from 13.59% to 13.74%), respectively. The *X_cr_* of the IMPLA was measured at 14.75%.

Table 4 summarizes the thermal properties of the samples, including the *T_g_*, *T_c_*, *T_m_*, and *T_cc_*. Across all compositions, specimens produced via IM exhibited a marginally higher degree of *X_cr_* compared to those fabricated by AM, a difference that may be attributed to the increased porosity observed in the AM samples [21].

Both AM and IM were found to exert only a limited influence on the *T_g_*, *T_c_*, and *T_m_* of the material. This observation is supported by several studies reporting no significant differences in the thermal properties between specimens produced via these two fabrication methods [21,87]. The DSC results obtained in this study exhibit a consistent agreement with previously reported findings in the literature [21,85,86,87].

#### 3.6.2. TGA Results

**Figure 11:**
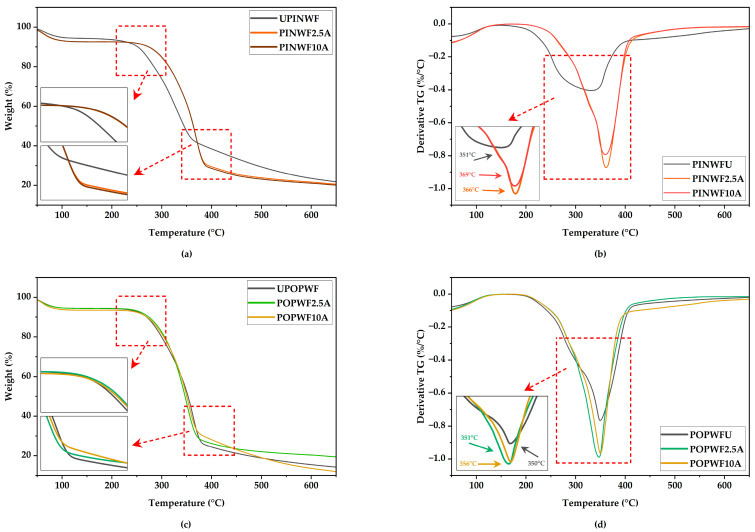
TGA and DTG curves of pine (**a**,**b**) and poplar (**c**,**d**) wood flour.

**Figure 12:**
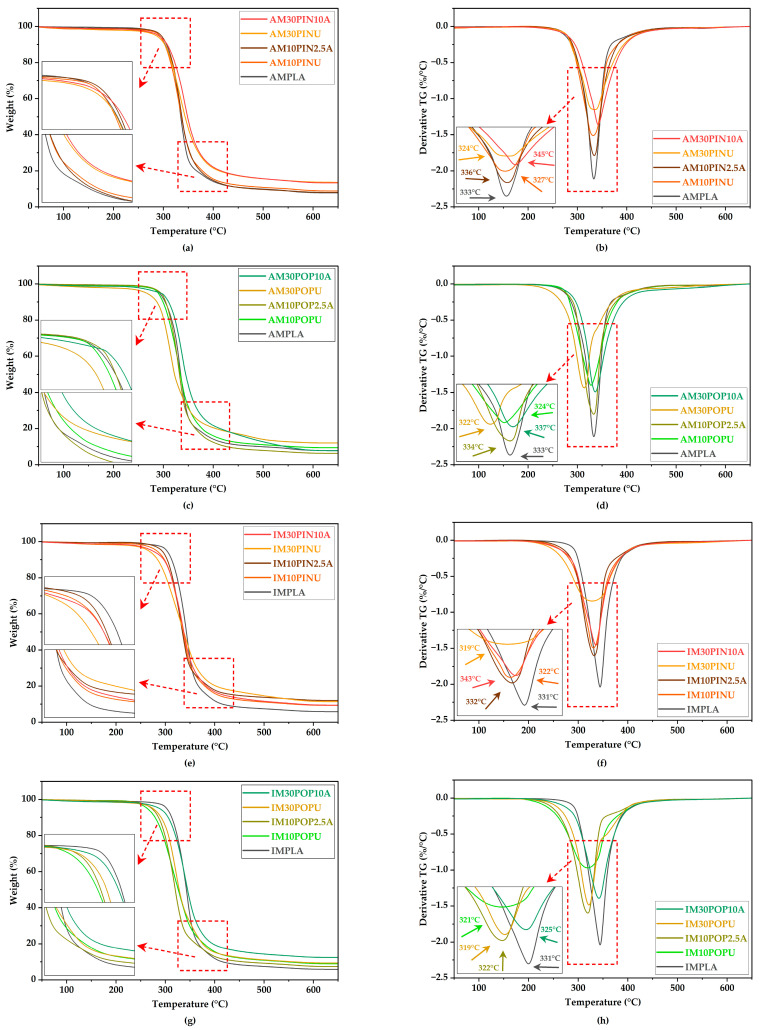
TGA and DTG curves of AM (**a**–**d**) and IM (**e**–**h**).

As illustrated in Figure 11 and Figure 12, TGA and derivative thermogravimetry (DTG) were employed under a nitrogen atmosphere to evaluate the thermal decomposition behavior and thermal stability of PLA, untreated and treated wood flour, as well as their reinforced biocomposites.

Due to their recyclability, thermoplastics require thermal degradation analysis to assess composite stability at high temperatures. TGA and DTG are used to determine their decomposition behavior under a nitrogen atmosphere [88].

All mass loss curves overlapped during the major degradation stage up to ~350 °C, indicating comparable T_onset_ and T_DTG peak_ values among the samples (Figure 11 and Figure 12; Table 5 and Table 6).

As the wood flour content in the biocomposites increased, a decrease in thermal degradation temperature was observed [10].

Furthermore, the maximum degradation temperatures identified in the DTG curves of the untreated wood flour-reinforced biocomposites were slightly lower than those of the control samples, AMPLA and IMPLA, which exhibited peak degradation at 332.95 °C and 330.56 °C, respectively.

During the second stage of thermal degradation, decomposition of the wood flour/PLA composites occurred between 200 °C and 400 °C, primarily due to the breakdown of hemicellulose, cellulose, pectin, and lignin. In the NaOH-treated samples, this stage began at 298.31 °C for AM30PIN10A, whereas the untreated counterpart (AM30PINU) exhibited an earlier onset at 285.47 °C. Similarly, in IM samples, degradation initiated at 286.66 °C for IM30PIN10A and at 280.72 °C for the untreated IM30PINU. A pronounced mass loss was observed in all formulations beyond 300 °C, marking the peak of thermal decomposition.

Consistent with previous studies, the TGA results confirm that alkaline treatment enhances the thermal stability of lignocellulosic particles, as evidenced by the modest elevation in thermal degradation onset temperatures observed in treated samples compared to their untreated counterparts [88,89].

Previous research has reported that PLA processed via IM exhibits reduced thermal stability compared to its 3D-printed counterparts. This reduction is presumed to result from polymer degradation induced by high shear forces encountered during pelletization and molding [82].

## 4. Conclusions

This study demonstrated the successful development of sustainable PLA-based biocomposites reinforced with untreated and alkali-treated wood flour derived from recycled Turkish red pine and white poplar wooden pallets. Biocomposite pellets and filaments were produced via twin-screw extrusion and subsequently fabricated into test specimens using both AM and IM. No processing anomalies such as die clogging, inconsistent flow, or thermal degradation were observed across either method, confirming good processability of the developed formulations.

Mechanical performance was significantly influenced by both wood flour surface treatment and processing route. IM samples containing 30 wt% alkali-treated Turkish red pine wood flour (10% NaOH) exhibited substantial improvements over untreated counterparts, with tensile strength increasing by 71.35%, flexural strength by 64.74%, and Shore D hardness by 2.62%. By contrast, the AM samples exhibited relatively lower improvements in tensile strength, flexural strength, and hardness, which can be attributed to inherent anisotropic behavior and insufficient inter-layer adhesion typical of the layer-by-layer printing process.

Physical and moisture resistance properties also benefited from alkali treatment. The AM sample with 10 wt% alkali-treated white poplar wood flour (10% NaOH) exhibited a 49.37% reduction in water absorption, indicating improved hydrophobicity and dimensional stability. This improvement is attributed to enhanced wood flour–matrix adhesion and decreased void content, as verified by SEM, which revealed more homogeneous wood flour dispersion and reduced interfacial porosity.

Thermal characterization through TGA showed that the alkali-treated wood flour retained stability up to 200 °C, confirming their compatibility with standard thermoplastic processing conditions. No significant differences were observed in thermal behavior between AM and IM samples, suggesting that the alkali treatment did not negatively impact the inherent thermal performance of the biocomposites.

Morphological and chemical analyses via SEM and FT-IR confirmed the presence of cellulose and hemicellulose structures, as well as improved interfacial bonding resulting from the alkali treatment. These structural enhancements are directly correlated with the observed mechanical and physical improvements.

Overall, this study provides a validated pathway for the use of industrial wood waste as a sustainable reinforcement source in PLA-based biocomposites. While untreated wood flour demonstrated moderate reinforcement, alkali-treated wood flour consistently outperformed in all tested parameters, supporting their viability for high-performance applications. However, when compared to other wood flour-reinforced biocomposites in literature the reported mechanical properties are comparable or superior, particularly under IM conditions, though a comprehensive comparative analysis.

In conclusion, alkali-treated wood flour recovered from waste wooden pallet offer a technically feasible, environmentally responsible, and industrially scalable reinforcement option for PLA composites, supporting the broader transition to circular economy, zero-waste material systems.

## Figures and Tables

**Table 1:** Filament and pellet formulations.

Sample Group	Production Methods		PLA (wt%)	Wood Flour (wt%)	NaOH (%)
	AM	IM			
	Sample ID				
Control	AMPLA	IMPLA	100	-	-
Pine	AM10PINU	IM10PINU	90	10	-
	AM10PIN2.5A	IM10PIN2.5A	90	10	2.5
	AM10PIN5A	IM10PIN5A	90	10	5
	AM10PIN10A	IM10PIN10A	90	10	10
	AM20PINU	IM20PINU	80	20	-
	AM20PIN2.5A	IM20PIN2.5A	80	20	2.5
	AM20PIN5A	IM20PIN5A	80	20	5
	AM20PIN10A	IM20PIN10A	80	20	10
	AM30PINU	IM30PINU	70	30	-
	AM30PIN2.5A	IM30PIN2.5A	70	30	2.5
	AM30PIN5A	IM30PIN5A	70	30	5
	AM30PIN10A	IM30PIN10A	70	30	10
Poplar	AM10POPU	IM10POPU	90	10	-
	AM10POP2.5A	IM10POP2.5A	90	10	2.5
	AM10POP5A	IM10POP5A	90	10	5
	AM10POP10A	IM10POP10A	90	10	10
	AM20POPU	IM20POPU	80	20	-
	AM20POP2.5A	IM20POP2.5A	80	20	2.5
	AM20POP5A	IM20POP5A	80	20	5
	AM20POP10A	IM20POP10A	80	20	10
	AM30POPU	IM30POPU	70	30	-
	AM30POP2.5A	IM30POP2.5A	70	30	2.5
	AM30POP5A	IM30POP5A	70	30	5
	AM30POP10A	IM30POP10A	70	30	10

**Table 2:** Particle size distribution results.

Wood Flour	D10	D50	D90
	μm		
Pine	155	346	737
Poplar	151	341	722

**Table 3:** Chemical composition of waste wood flour [27].

Waste Wood Flour		Holocellulose		Lignin	Extractive Substance	Ash	Moisture Content	
		Alpha Cellulose	Hemicellulose					
Raw	Pine	47.45	23.83	27.36	0.61	0.75	11.25	Ref. [27]
	Poplar	48.11	29.26	21.01	1.08	0.54	12.33	
2.5% NaOH	Pine	53.62	18.64	26.52	0.56	0.68	11.04	Present study
	Poplar	56.49	22.37	19.76	0.93	0.45	11.97	
10% NaOH	Pine	64.94	12.75	21.33	0.41	0.57	10.53	
	Poplar	65.87	17.89	15.01	0.87	0.36	11.28	

Note: All numerical values presented in the table are expressed as percentages (%).

**Table 4:** DSC analysis values of AM and IM biocomposites, compared to neat PLA.

Sample ID	*T_g_* (°C)	*T_c_* (°C)	*T_m_* (°C)	Δ*H_m_* (J.g^−1^)	*T_cc_* (°C)	Δ*H_cc_* (J.g^−1^)	*X_cr_* (%)
AMPLA	65.71	99.14	151.51	21.59	82.61	8.20	14.40
AM10PINU	64.87	97.76	150.33	21.06	82.60	7.24	13.37
AM10PIN2.5A	65.25	97.93	151.02	21.49	82.66	7.50	13.54
AM30PINU	63.47	93.70	148.56	20.17	82.29	6.00	10.67
AM30PIN10A	63.48	94.02	148.70	20.28	83.79	6.02	10.73
AM10POPU	64.23	96.82	150.16	20.96	80.78	7.18	13.33
AM10POP2.5A	65.71	98.66	151.25	21.17	82.83	7.22	13.50
AM30POPU	63.20	94.39	146.30	19.92	80.69	5.95	10.51
AM30POP10A	64.55	96.72	148.81	20.22	82.31	6.23	10.53
IMPLA	63.12	93.23	151.08	22.08	82.17	8.36	14.75
IM10PINU	61.63	88.63	149.03	21.63	82.11	7.44	13.73
IM10PIN2.5A	62.27	89.06	150.22	21.80	82.58	7.49	13.85
IM30PINU	60.05	86.75	147.58	20.34	82.05	6.12	10.70
IM30PIN10A	61.08	87.11	147.63	20.76	83.74	6.39	10.82
IM10POPU	61.16	88.84	149.09	21.46	80.03	7.41	13.59
IM10POP2.5A	62.40	89.99	150.68	21.64	82.26	7.45	13.74
IM30POPU	61.09	88.52	147.42	20.26	80.01	6.11	10.65
IM30POP10A	62.98	89.21	147.61	20.53	80.79	6.24	10.76

**Table 5:** Results obtained from the TGA analysis of wood flour.

Sample ID	T_onset_ (°C)	T_max_ (°C)	T_offset_ (°C)
UPINWF	265.42	351.12	381.02
UPIN2.5A	266.25	365.94	382.27
UPIN10A	269.64	369.12	388.03
UPOPWF	263.92	350.34	380.15
UPOP2.5A	265.24	351.11	381.33
UPOP10A	266.00	356.04	383.39

**Table 6:** Results obtained from the TGA analysis of biocomposite.

Sample ID	T_onset_ (°C)	T_max_ (°C)	T_offset_ (°C)
AMPLA	294.59	332.95	391.13
AM10PINU	290.11	327.29	389.35
AM10PIN2.5A	296.07	336.22	390.23
AM30PINU	285.47	324.33	385.74
AM30PIN10A	298.31	345.51	387.68
AM10POPU	288.08	324.36	388.49
AM10POP2.5A	296.00	334.17	389.91
AM30POPU	284.88	322.47	383.34
AM30POP10A	297.53	337.06	384.72
IMPLA	293.35	330.56	384.03
IM10PINU	282.24	321.68	382.08
IM10PIN2.5A	285.75	332.00	383.44
IM30PINU	280.72	319.32	380.18
IM30PIN10A	286.66	342.78	381.14
IM10POPU	281.12	320.70	380.93
IM10POP2.5A	282.35	321.83	382.22
IM30POPU	279.11	318.90	378.72
IM30POP10A	286.47	325.12	379.83

## Data Availability

Data are available in this article.
